# Final analyses of the prospective controlled trial on the efficacy of uracil and tegafur/leucovorin as an adjuvant treatment for stage II colon cancer with risk factors for recurrence using propensity score-based methods (JFMC46-1201)

**DOI:** 10.1007/s10147-024-02565-5

**Published:** 2024-06-04

**Authors:** Yutaka Ogata, Sotaro Sadahiro, Kazuhiro Sakamoto, Takashi Tsuchiya, Takao Takahashi, Hiroki Ohge, Toshihiko Sato, Ken Kondo, Hideo Baba, Michio Itabashi, Masataka Ikeda, Madoka Hamada, Kiyoshi Maeda, Hiroyuki Masuko, Keiichi Takahashi, Mitsuo Kusano, Ichinosuke Hyodo, Junichi Sakamoto, Masataka Taguri, Satoshi Morita

**Affiliations:** 1 Department of Surgery, Kurume University School of Medicine, 67 Asahi-Machi, Kurume, Fukuoka 830-0011 Japan; 2 Department of Surgery, Tokai University, 143 Shimokasuya, Isehara, Kanagawa 259-1193 Japan; 3 Department of Coloproctological Surgery, Juntendo University, 2-1-1 Hongo, Bunkyo-Ku, Tokyo, 113-8421 Japan; 4 Department of Surgery, Sendai City Medical Center, 5-22-1 Tsurugaya, Miyagino-Ku, Sendai, Miyagi 983-0824 Japan; 5 Department of Digestive Surgery, Gifu University Hospital, 1-1 Yanagido, Gifu, 501-1194 Japan; 6 Department of Surgery, Seino Kosei Hospital, 293-1 Shimoiso Ono-Cho, Ibi-Gun, Gifu, 501-0532 Japan; 7 Department of Infectious Diseases, Hiroshima University Hospital, 1-2-3 Kasumi, Minami-Ku, Hiroshima, 734-8551 Japan; 8 Department of Surgery, Yamagata Prefectural Central Hospital, 1800 Aoyagi, Yamagata, 990-2292 Japan; 9 Department of Surgery, Nagoya Medical Center, 4-1-1 Sannomaru, Naka-Ku, Nagoya, Aichi 460-0001 Japan; 10 Department of Gastroen- Terological Surgery, Kumamoto University, 1-1-1 Honjo, Chuo-Ku, Kumamoto, 860-8556 Japan; 11 Department of Surgery, Division of Inflammatory Bowel Disease Surgery, Tokyo Women’s Medical University, 8-1 Kawada-Cho, Shinjuku-Ku, Tokyo, 162-8666 Japan; 12 Department of Gastroenterological Surgery, Division of Lower GI, Hyogo Medical University, 1-1 Mukogawa-Cho, Nishinomiya, Hyogo 663-8501 Japan; 13 Department of Gastrointestinal Surgery, Kansai Medical University Hospital, 2-3-1 Shinmachi Hirakata, Osaka, 573-1191 Japan; 14 Department of Gastroenterological Surgery, Osaka Metropolitan University, 1-4-3 Asahimachi, Abeno-Ku, Osaka, 545-8585 Japan; 15 Department of Surgery, Nikko Memorial Hospital, 1-5-13 Shintomi-Cho, Muroran, Hokkaido 051-8501 Japan; 16 Tokyo Metropolitan Health and Hospitals Corporation Ohkubo Hospital, 2-44-1 Kabuki-Cho, Shinjuku-Ku, Tokyo, 160-8488 Japan; 17 Department of Physical Medicine, Yoichi Hospital, 19-1-1 Kurokawa-Cho Yoichi, Hokkaido, 046-0003 Japan; 18 Department of Gastrointestinal Medical Oncology, National Hospital Organization Shikoku Cancer Center, 160 Kou, Minamiumemoto, Matsuyama, Ehime 791-0280 Japan; 19 Tokai Central Hospital, 4-6-2 Sohara Higashijima-Cho, Kakamigahara, Gifu, 504-8601 Japan; 20 Department of Health Data Science, Tokyo Medical University, 6-1-1 Shinjuku, Shinjuku-Ku, Tokyo, 160-8402 Japan; 21 Department of Biomedical Statistics and Bioinformatics, Kyoto University, 54 Kawahara-Cho, Shogoin, Sakyo-Ku, Kyoto, 606-8507 Japan

**Keywords:** Adjuvant chemotherapy, Colon cancer, High-risk stage II, Propensity score, Risk factor, Tegafur, Uracil, Leucovorin, Inverse probability of treatment-weighting

## Abstract

**Background:**

The efficacy of adjuvant chemotherapy for high-risk stage II colon cancer (CC) has not been well established. Using propensity score matching, we previously reported that the 3-year disease-free survival (DFS) rate was significantly higher in patients treated with uracil and tegafur plus leucovorin (UFT/LV) against surgery alone. We report the final results, including updated 5-year overall survival (OS) rates and risk factor analysis outcomes.

**Methods:**

In total, 1902 high-risk stage II CC patients with T4, perforation/penetration, poorly differentiated adenocarcinoma/mucinous carcinoma, and/or < 12 dissected lymph nodes were enrolled in this prospective, non-randomized controlled study based on their self-selected treatment. Oral UFT/LV therapy was administered for six months after surgery.

**Results:**

Of the 1880 eligible patients, 402 in Group A (surgery alone) and 804 in Group B (UFT/LV) were propensity score-matched. The 5-year DFS rate was significantly higher in Group B than in Group A (P = 0.0008). The 5-year OS rates were not significantly different between groups. The inverse probability of treatment weighting revealed significantly higher 5-year DFS (P = 0.0006) and 5-year OS (P = 0.0122) rates in group B than in group A. Multivariate analyses revealed that male sex, age ≥ 70 years, T4, < 12 dissected lymph nodes, and no adjuvant chemotherapy were significant risk factors for DFS and/or OS.

**Conclusion:**

The follow-up data from our prospective non-randomized controlled study revealed a considerable survival advantage in DFS offered by adjuvant chemotherapy with UFT/LV administered for six months over surgery alone in individuals with high-risk stage II CC.

**Trial registration:**

Japan Registry of Clinical Trials: jRCTs031180155 (date of registration: 25/02/2019), UMIN Clinical Trials Registry: UMIN000007783 (date of registration: 18/04/2012).

## Introduction

Colorectal cancer (CRC) is the third most common malignancy and second most frequent cause of cancer-related mortality worldwide [1]. According to the American Society of Clinical Oncology, the National Comprehensive Cancer Network, and the European Society for Medical Oncology, it is recommended that patients with stage II colon cancer (CC) and a high risk of recurrence receive adjuvant chemotherapy [2–5]. According to the Japanese Society for Cancer of the Colon and Rectum’s clinical practice guidelines, adjuvant chemotherapy is recommended for stage II colorectal cancer patients who have a high likelihood of recurrence [6]. Nevertheless, while the advantages of adjuvant chemotherapy for stage III CC are well-established, its benefits for stage II CC remain uncertain due to the absence of conclusive data from randomized controlled studies [7, 8]. Additionally, the risk factors for recurrence in stage II CC remain to be fully established [9–11]. Subset analyses of the MOSAIC study revealed no statistically significant improvement in the efficacy of adjuvant therapy with the addition of oxaliplatin to 5-fluorouracil (FL) for stage II CC, regardless of the presence or absence of risk factors for recurrence [12].

Oral uracil and tegafur plus leucovorin (UFT/LV) have demonstrated effectiveness and safety comparable to those of intravenous 5-fluorouracil/LV in individuals with stage II/III cancer in Japan [13, 14]. A definitive advantage of oral UFT/LV as adjuvant chemotherapy over intravenous 5-fluorouracil/LV is increased convenience [14]. We conducted a multicenter prospective trial with two arms, one of which was non-randomized. Patients diagnosed with stage II CC were given the option to either undergo surgery alone or surgery followed by oral UFT/LV. Propensity score (PS) matching [15, 16] was employed to mitigate confounding biases in comparing between the groups. Due to an insufficient number of patients enrolled in the randomized arm, it was excluded from the analyses. In this prospective study utilizing PS matching, a 6-month course of oral UFT/LV therapy demonstrated an enhancement in the 3-year disease-free survival (DFS) rate in comparison to surgery alone (UFT/LV = 80.9%, surgery alone = 74.0%; hazard ratio = 0.64; 95% CI 0.50 to 0.83; P = 0.0006). Furthermore, it exhibited an acceptable safety profile among patients who underwent curative-intent resection for stage II CC [17]. Additionally, multivariate analyses indicated that oral UFT/LV postoperative therapy for 6 months was a significant independent prognostic factor for DFS [18]. Nevertheless, the overall survival (OS) data provided in the previous study were preliminary. Herein, we present the final results of the study, including the updated 5-year DFS and OS rates, and report the outcomes of risk factor analyses for DFS and OS.

## Patients and methods

The specifics of the study protocol are available in our previous report [19].

### Patients

Eligible patients, aged 20–80 years, with histologically confirmed stage II colon adenocarcinoma, met at least one of the following criteria: T4 disease, perforation/penetration, poorly differentiated adenocarcinoma/mucinous carcinoma, or < 12 dissected lymph nodes. Additionally, they had undergone R0 resection, possessed an Eastern Cooperative Oncology Group performance status of 0/1, and were able to commence the study treatment with oral UFT/LV within eight weeks post-surgery.

### Ethics approval and consent to participate

Prior to enrollment, written informed consent was obtained from all patients. The multi-institutional study, designated as JFMC46-1201, received approval from the Certified Review Board (CRB3180009) of the National Cancer Center Hospital East and was conducted in compliance with the Declaration of Helsinki.

### Allocation to treatment

This prospective, non-randomized controlled study was based on patients’ self-selected treatments: surgery alone (Group A) or surgery followed by UFT/LV (Group B). Patients in Group A underwent blood tests every 3 months and chest and abdominal computed tomography examinations every 6 months for up to 5 years or until confirmation of recurrence, occurrence of other malignancies, or death. Colonoscopy was also performed 1 and 3 years after surgery. Further details regarding the UFT/LV treatment can be found in our previous report [17]. Following the completion of the 6-month UFT/LV therapy, patients were monitored according to the same schedule used in Group A, with the exception that adverse events (AEs) were monitored from the start of the UFT/LV therapy until 28 days after the final dose.

### Endpoints

The primary endpoint of the study was to evaluate DFS, which was the period between the registration date and the first occurrence of secondary cancer, recurrence, or death form any cause. The secondary endpoints were OS, which was the duration from the registration date to death from any cause, as well as the frequency of AEs based on their severity. The prognostic indicators for DFS and OS were determined through a multivariate analysis.

### Statistical analysis

The target sample size was projected to be 1,715 patients, with a 1:2 ratio between Groups A and B [19]. To assess survival benefits, 1:2 PS matching was employed to mitigate confounding biases when comparing Groups A and B [15, 16]. Prespecified potential confounding factors for PS estimation included sex, age (≥ 70 or < 70 years), T4 disease, bowel perforation, poorly differentiated adenocarcinoma, mucinous carcinoma, number of dissected lymph nodes (≥ 12 or < 12), and number of participating patients at each institution (≥ 5 or < 5). Further details regarding the PS matching method can be found in our previous reports [17, 19]. The Kaplan–Meier method was utilized to estimate the 5-year DFS and OS rates in each group, with 95% confidence intervals calculated using Greenwood’s formula.

Analyses were additionally conducted utilizing inverse probability of treatment weighting (IPTW) based on the estimated PSs [20]. For IPTW analysis, standard errors were calculated using a robust sandwich variance estimator [21]. Multivariate analyses of prognostic factors, including age, sex, tumor location, depth of tumor invasion, perforation/penetration, poorly differentiated component, mucinous component, number of examined lymph nodes, number of participating patients at each institution, tumor location, and postoperative adjuvant chemotherapy, were executed using Cox proportional hazard regression via the backward elimination approach, with a significance level of P = 0.05 for exclusion. All statistical analyses were performed using SAS version 9.4 (SAS Institute, Cary, NC, USA).

## Results

### Patient characteristics before and after PS matching

A total of 1902 patients from 321 Japanese institutions were enrolled in this study between May 2012 and April 2016. Of these, 1880 patients were deemed eligible for the non-randomized study, comprising 641 patients in Group A and 1239 patients in Group B. Following 1:2 propensity score matching, 402 patients in Group A and 804 patients in Group B were successfully matched, with no significant differences observed in the eight confounding factors between the groups, as detailed in a prior report [17]. The standardized difference for all confounding factors was < 0.08.

### Survival outcomes in the PS-matched population

**Fig. 1:**
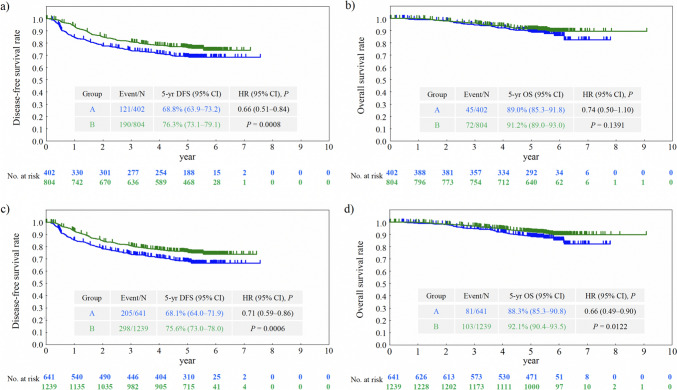
Disease-free and overall survival in patients with resected high-risk stage II colon cancer. **a** Disease-free and **b** overall survival in the propensity score-matched groups. **c** Disease-free and **d** overall survival in the inverse probability of treatment weighting groups. Group A (blue): surgery alone; Group B (green): surgery followed by UFT/LV treatment. *CI* confidence interval, *DFS* disease-free survival, *HR* hazard ratio, *OS* overall survival, *UFT/LV* uracil, and tegafur plus leucovorin. *P* values were obtained using the log-rank test

As of the data cut-off date on January 13, 2022, the median follow-up time for DFS in Groups A and B was 60.5 months (interquartile range [IQR], 59.9–63.1 months) in the PS-matched population. The 5-year DFS rate was significantly higher in Group B (76.3% [73.1–79.1%]) than in Group A (68.8% [63.9–73.2%]) (HR = 0.66 [0.51–0.84]; P = 0.0008) (Fig. 1a). The median follow-up time for OS in Groups A and B was 61.3 months (IQR, 60.3–65.3 months) in the PS-matched population. No significant difference was observed in the 5-year OS rate between Group A (89.0% [85.3–91.8%]) and Group B (91.2% [89.0–93.0%]) (HR = 0.74 [0.50–1.10]; P = 0.1391) (Fig. 1b).

### Events in the PS-matched population

There were 121 (30.1%) and 190 (23.6%) DFS events in Groups A and B, respectively. Recurrence of primary CC occurred in 75 patients (18.7%) in Group A commonly in the lung and liver, and in 127 patients (15.8%) in Group B, most commonly in the peritoneum and liver. The median time to recurrence was significantly longer in Group B (17.3 months; range, 1.8–59.8 months) than in Group A (8.7 months; range, 2.0–52.6 months) (P < 0.0001). Moreover, 45 (11.2%) and 72 (9.0%) OS events (all deaths) occurred in Groups A and B, respectively (Table 1).

**Table 1:** Summary of disease-free survival and overall survival events

	Surgery alone (Gr-A)	UFT/LV (Gr-B)
	*n* = 402	*n* = 804
**Disease-free survival events**	121 (30.1)	190 (23.6)
Recurrence	75 (18.7)	127 (15.8)
Sites (multiple selections made)		
Liver	24 (6)	37 (4.6)
Lung	26 (6.5)	30 (3.7)
Local	12 (3)	27 (3.4)
Anastomosis	5 (1.2)	15 (1.9)
Regional lymph node	0 (0)	4 (0.5)
Other	7 (1.7)	11 (1.4)
Lymph nodes other than regional lymph nodes	5 (1.2)	8 (1)
Peritoneum	18 (4.5)	41 (5.1)
Other	4 (1)	11 (1.4)
Uterus	0 (0)	1 (0.1)
Ovary	2 (0.5)	3 (0.4)
Other	2 (0.5)	7 (0.9)
Secondary cancer	41 (10.2)	55 (6.8)
Death	5 (1.2)	8 (1)
**Overall survival events**	45 (11.2)	72 (9)

Values presented as *n* (%)

The first disease-free survival event experienced by each patient was exhibited. Gr-A: patients underwent surgery alone; Gr-B: patients underwent surgery followed by UFT/LV

*UFT/LV* Uracil and tegafur plus leucovorin

### Survival outcomes in the IPTW population

The patients’ background characteristics were well balanced between Group A (n = 641) and Group B (n = 1239), as shown in a previous report [17]. The median follow-up time for DFS was 60.5 months (IQR, 60.0–63.0 months) and the 5-year DFS rate (95% CI) was significantly higher in Group B (75.6% [73.0–78.0%]) than in Group A (68.1% [64.0–71.9%]) (HR = 0.71 [0.59–0.86]; P = 0.0006) (Fig. 1c). The median follow-up time for OS was 61.3 months (IQR, 60.3–65.2 months), and the 5-year OS rate was significantly higher in Group B (92.1% [90.4–93.5%]) than in Group A (88.3% [85.3–90.8%]) (HR = 0.66 [0.49–0.90]; P = 0.0122) (Fig. 1d).

**Fig. 2:**
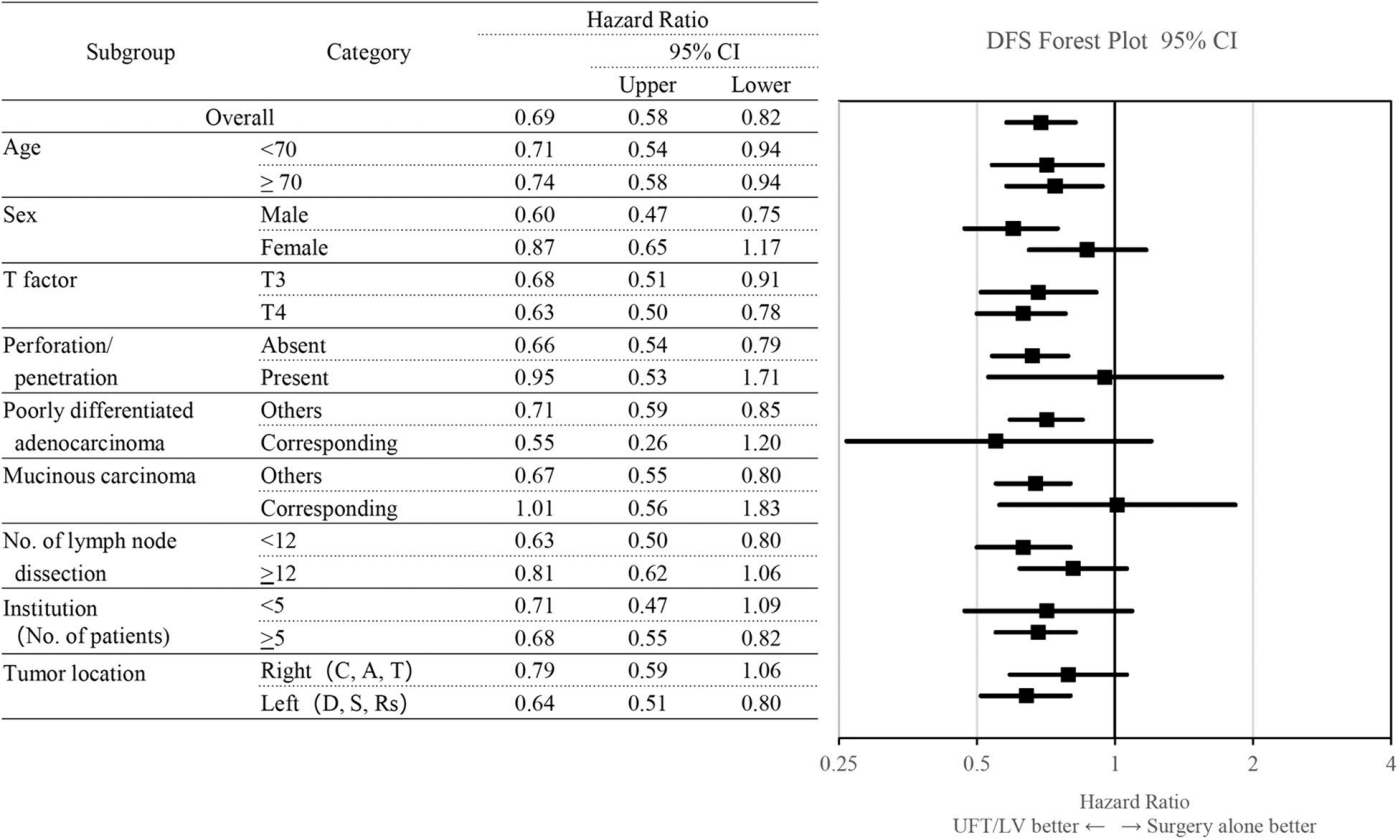
Hazard ratios and 95% CIs for DFS events in patients treated with surgery followed by UFT/LV treatment compared with those in patients treated with surgery alone according to baseline prognostic factors (inverse probability of treatment-weighting groups). Analyses were conducted using a Cox regression model. *CI* confidence interval, *DFS* disease-free survival, *UFT/LV* uracil and tegafur plus leucovorin

**Fig. 3:**
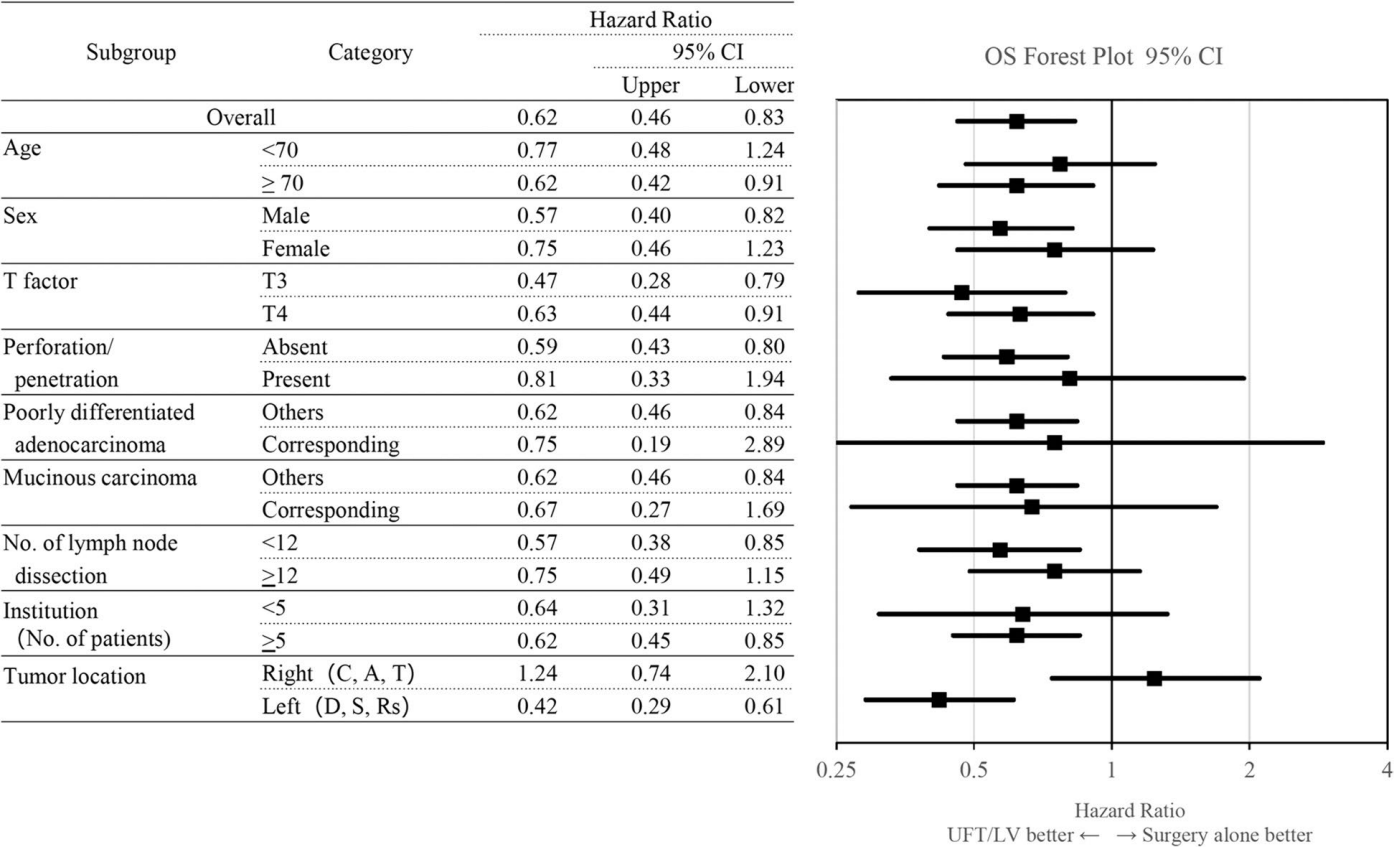
Hazard ratios and 95% CIs for death in patients treated with surgery followed by UFT/LV treatment compared with those in patients treated with surgery alone according to baseline prognostic factors (inverse probability of treatment weighting groups). Analyses were conducted using a Cox regression model. *CI* confidence interval, *OS* overall survival, *UFT/LV* uracil and tegafur plus leucovorin

Subgroup analyses were conducted to ascertain prognostic factors for DFS and OS within the IPTW population. Regarding both DFS (Fig. 2) and OS (Fig. 3), the estimation of hazard ratios and 95% CIs showed that the potential benefit from UFT/LV chemotherapy could not be ruled out across most subgroups delineated by baseline prognostic factors. Additionally, a significant benefit from UFT/LV was established in several subgroups.

In the multivariate analysis, the Cox proportional hazards model identified the following statistically significant prognostic factors for DFS and OS: age, sex, T factor, number of dissected lymph nodes, and postoperative adjuvant chemotherapy. The effects of the UFT/LV treatment on these factors were consistent (Table 2).

**Table 2:** Multivariate analysis for prognostic factors for disease-free survival (DFS) and overall survival (OS)

Factor	Category	IPTW population			
		DFS		OS	
		Hazard ratio	*P* value	Hazard ratio	*P *value
		Estimate		Estimate	
Treatment group	(UFT-LV / surgery alone)	0.71	0.0002*	0.65	0.0043*
Age	(≥ 70 yr / < 70 yr)	1.28	0.0077*	1.52	0.0065*
Sex	(female/male)	0.79	0.0083*	0.66	0.0059*
T factor	(T4/T3)	2.27	< 0.0001*	3.20	< 0.0001*
No. of lymph node dissection	(< 12/ ≥ 12)	1.72	< 0.0001*	2.26	< 0.0001*

Hazard ratios were calculated using Cox proportional hazard regression by backward elimination method

^*^P < 0.05

*IPTW* inverse probability of treatment weighting

### Treatment with UFT/LV and safety

The details of treatment with UFT/LV and its safety profile can be found in our previous report [17]. The 6-month oral UFT/LV therapy was safe and well tolerated as postoperative adjuvant chemotherapy.

## Discussion

Based on the 3-year DFS improvement previously reported in this trial [17], we suggested that adjuvant UFT/LV therapy should be considered beneficial after surgery for stage II CC with a high risk of recurrence, such as T4 disease, perforation/penetration, poorly differentiated adenocarcinoma/mucinous carcinoma, and/or < 12 dissected lymph nodes. The Adjuvant Colon Cancer Endpoints (ACCENT) meta-analysis of adjuvant studies, which was performed before the approval of oxaliplatin and irinotecan for advanced disease, demonstrated that 3-year DFS is an excellent predictor of 5-year OS [22] and could be an appropriate primary endpoint for adjuvant studies in CC. Based on these findings, 3-year DFS was recognized by the US Food and Drug Administration as a primary endpoint for studies on adjuvant CC.

In this final analysis, encompassing an extended 5-year DFS period among the propensity score-matched patients, a statistically significant overall relative risk reduction of 34% for DFS events—encompassing recurrence and secondary cancer—was observed in favor of UFT/LV therapy, thus affirming the benefit previously reported 3-year DFS. However, no significant disparity was noted in the extended 5-year OS between the UFT/LV therapy and surgery-alone groups. This outcome suggests that while the 3-year DFS advantage persisted, it did not translate into a corresponding 5-year OS benefit in this study. Further analysis in ACCENT also indicated that patients with stage II CC had more prolonged survival following tumor recurrence than those with stage III CC [23]. This may explain why the significantly improved 3-year DFS in the present study did not translate into a 5-year OS benefit. The rate of 5-year OS events (all deaths) was extremely low for surgery alone (11.2%) and UFT/LV therapy (9.0%), with remarkably high 5-year OS rates of 89.0% and 91.2%, respectively. Recent improvements in chemotherapy, such as molecular targeted therapy with bevacizumab and cetuximab, as well as aggressive surgery for metastases, may have extended the survival time for patients with CC who experience postoperative recurrence [24, 25]. This possibly contributes to the prolonged survival observed after recurrence, which diminished the relationship between the 3-year DFS and 5-year OS rates in this study, which utilized a population matched by PS.

The rate of primary cancer recurrence within five years post-surgery was 18.7% (75 patients) among those treated with surgery alone and 15.8% (127 patients) among those receiving UFT/LV therapy. These figures remained consistent with those observed at the 3-year follow-up, as most postoperative relapses occurred within the initial three years. Nevertheless, 60% of patients (45 out of 75) in the surgery-alone group and 57% of patients (72 out of 127) in the UFT/LV group who experienced postoperative recurrence were still alive after five years of follow-up (data not shown). Consequently, no significant difference was observed in the 5-year OS between the surgery-alone and UFT/LV groups. Hence, an extended follow-up duration is warranted to detect potential improvements in OS following adjuvant chemotherapy, given the favorable overall prognosis of stage II CC patients. In the analysis of ACCENT, de Gramont et al. concluded that future trials evaluating adjuvant chemotherapy for stage II CC should incorporate a minimum follow-up of 6–7 years to determine a clinically relevant survival benefit [26]. Considering these findings, we believe that patients from the PS-matched population in the present study require extended follow-up for more than seven years before conclusions regarding the OS benefits of UFT/LV can be drawn.

IPTW patients treated with UFT/LV had significantly improved 5-year OS rates. This improvement could be attributed to the higher statistical power achieved by including all the patients (n = 1,880). Additionally, upon comparing hazard ratios and 95% CIs for death between IPTW patients treated with UFT/LV and those treated with surgery alone, based on baseline prognostic factors, the potential benefit of UFT/LV chemotherapy was not ruled out in the majority of defined subgroups. Furthermore, multivariate analysis revealed that UFT/LV adjuvant chemotherapy was a significant independent prognostic factor for improved OS.

Collectively, these data suggest that postoperative adjuvant chemotherapy using UFT/LV may be helpful in patients with stage II CC with risk factors for recurrence, such as T4 disease, perforation/penetration, poorly differentiated adenocarcinoma/mucinous carcinoma, and/or < 12 dissected lymph nodes. Multivariate analyses also suggested that sex, age, T4 disease, and number of dissected lymph nodes were significant risk factors for both DFS and OS in stage II CC. Moreover, based on multivariate analyses, only T4 disease, perforation/penetration, and the number of dissected lymph nodes were significant risk factors for relapse-free survival (RFS) (data not shown). Age and sex were identified as significant risk factors for both DFS and OS, considering the inclusion of secondary cancer and death due to multiple diseases. The observed results regarding recurrence risk factors may be attributed to the absence of D3 lymph node dissection, which involves comprehensive dissection of paracolic and mesenteric lymph nodes in most cases involving perforation or penetration [18]. This suggests a robust correlation between perforation/penetration and the number of dissected lymph nodes (< 12). Consequently, in line with the clinical guidelines published by ASCO [3] and ESMO [4], the depth of tumor invasion (T4) and the number of examined lymph nodes (< 12) were deemed reliable risk factors for recurrence.

Studies involving patients with stage II/III or III CC have shown that 5-fluorouracil (with or without oxaliplatin or LV), capecitabine (with or without oxaliplatin), and UFT/LV are effective adjuvant chemotherapies. However, the efficacy of these therapies in patients with stage II CC is inconsistent [12, 13, 27–29]. In a recent nationwide study and survey based on IPTW, long-term quality of life (QoL) diminished with adjuvant chemotherapy, with oxaliplatin-induced neurotoxicity closely associated with decreased QoL in patients with CC [30]. Therefore, we were particularly interested in adjuvant chemotherapy, which uses oral agents that are more convenient than oxaliplatin-based regimens, especially in patients with stage II CC. In this study, we applied PS matching to adjust for confounding variables to assess the effects of UFT/LV. An increasing number of studies have employed this analytical approach, and our findings suggest that it is indeed a suitable statistical method for achieving comparable distributions of observed baseline covariates. Currently, conducting a large-scale randomized clinical trial of adjuvant therapy for stage II CC with a long-term follow-up OS of > 7 years, as described above, is exceptionally challenging. Hence, we anticipate that data derived from meta-analyses and non-randomized controlled studies, analyzed using PS matching, will offer both statistically and clinically significant insights, especially concerning adjuvant therapy for stage II CC.

Despite the substantial contributions to the existing literature, our study does have some limitations. Although PS matching and IPTW were used to adjust for risk factors, some risk factors, such as microsatellite instability (MSI) [31, 32], were not considered; for instance, MSI status is known to influence the benefit of postoperative adjuvant chemotherapy with fluoropyrimidine alone in colon CC [31]. We also excluded lymphatic, vascular, and perineural invasion as risk factors due to potential inconsistencies in evaluation among the participating institutions. Further studies are necessary to validate the present results concerning the risk factors used for adjustment.

## Conclusion

The outcomes of our prospective, non-randomized controlled study indicate that six months of adjuvant chemotherapy with oral UFT/LV leads to substantial survival benefits for patients with stage II CC who exhibit at least one of the following characteristics: T4 disease, perforation/penetration, poorly differentiated adenocarcinoma/mucinous carcinoma, or < 12 dissected lymph nodes. Our findings may inform tailored treatment strategies by emphasizing the importance of individual risk assessment in post-surgical care. Clinicians must judiciously evaluate the benefits of adjuvant therapy against its impact on patients’ QoL, especially given the varying responses based on risk factors such as T4 disease, perforation/penetration, and lymph node involvement. Our evidence supports a more personalized approach to treating stage II CC, guiding clinicians in making informed decisions that balance efficacy with patient well-being.

## Data Availability

The datasets used and/or analyzed during the current study are available from the corresponding author upon reasonable request.
